# COVID Vaccine Rollout for Older Adults in Japan

**DOI:** 10.1093/geroni/igab046.712

**Published:** 2021-12-17

**Authors:** Tadashi Wada

**Affiliations:** Irahara Primary Care Hospital, Chiba, Chiba, Japan

## Abstract

It has just started in Japan. We will provide detailed information later. The symposium has experts from 8 countries. We will use an interview and dialog format, instead of presentations.

